# Psychological Background of Sexual Dysfunctions – a Comparative Study on Hungarian and Spanish Samples

**DOI:** 10.1192/j.eurpsy.2024.776

**Published:** 2024-08-27

**Authors:** Z. G. Pintér-Eszenyei, S. Kató, Á. Hőgye-Nagy, A. K. Csinády, M. A. Rando Hurtado, A. Szemán-Nagy

**Affiliations:** ^1^Faculty of Humanities, Institute of Psychology, University of Debrecen, Debrecen, Hungary; ^2^Faculty of Psychology and Speech Therapy, University of Málaga, Málaga, Spain

## Abstract

**Introduction:**

Sexual dysfunctions are prevalent issues affecting individuals’ sexual well-being and relationships. These conditions encompass a range of difficulties in sexual functioning, from desire and arousal to orgasm and pain. Psychological factors, such as dysfunctional beliefs about sexuality, play a significant role in the development and perpetuation of sexual dysfunctions (Nobre, Pinto-Gouveia, 2006; Nobre, Pinto-Gouveia, 2008). Additionally, personality traits, particularly those associated with the Dark Triad (Machiavellianism, narcissism, and psychopathy), have been suggested as potential protective factors to sexual problems, probably in interaction with sexual assertiveness and a wider experience in sexual behavior (Pilch, Smolorz, 2019).

**Objectives:**

This study investigates the interplay between sexual dysfunctions, sexual dysfunctional beliefs, and Dark Triad personality traits, and compares the differences and similarities in the two different cultural (Hungarian and Spanish) samples.

**Methods:**

Both samples were collected online by sharing the questionnaires on various platforms. Apart from the demographic and sexuality related background questions (age, sex, gender, sexual orientation, sexual lifestyle, etc.) our set of questionnaires included the Arizona Sexual Experience Scale (ASEX), Sexual Dysfunctional Beliefs Questionnaire (SDBQ, Male and Female Version) and The Short Dark Triad Questionnaire (SD3).

**Results:**

The Hungarian sample consists of 465 participants, the Spanish of 215. However, the processing of the data is still underway, our preliminary results show, that there is a connection between the number of dysfunctional beliefs and occurrence of sexual dysfunctions. Just like Dark Triad traits seem to have negative correlation with dysfunctions.

**Conclusions:**

Our research gives an opportunity to a better understanding of the psychological background of sexual dysfunctions. By taking in consideration the relationship between dysfunctional beliefs and said disorders, professionals can optimize sexual education to aid the prevention of them. Nevertheless, our findings can help the practice of psychotherapy in finding more advanced treatments, thus improving individuals’ overall sexual, and general well-being.

**Disclosure of Interest:**

None Declared

